# Supplementary material for: Cranial Mandibular Fibrosis Syndrome in Adult Farmed Rainbow Trout Oncorhynchus mykiss
Source: Pathogens. 2021 Apr 30;10(5):542. doi: 10.3390/pathogens10050542 (PMC8145062; doi:10.3390/pathogens10050542)
Supplement: Supplementary file 1 [file pathogens-10-00542-s001.zip › pathogens-1166242-SI.pdf]

pathogens

MDPI

Supplementary Materials

a) Jaw

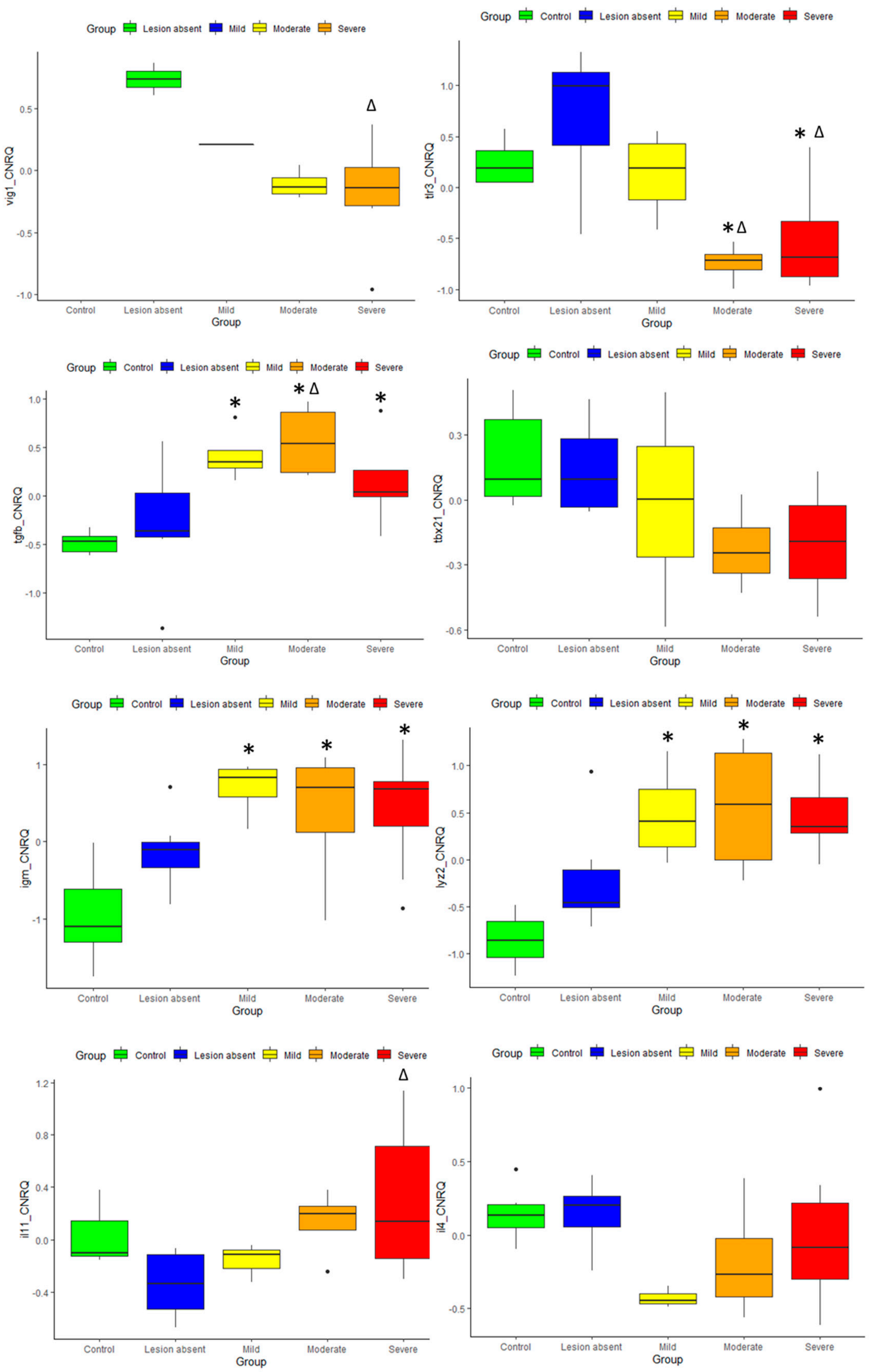

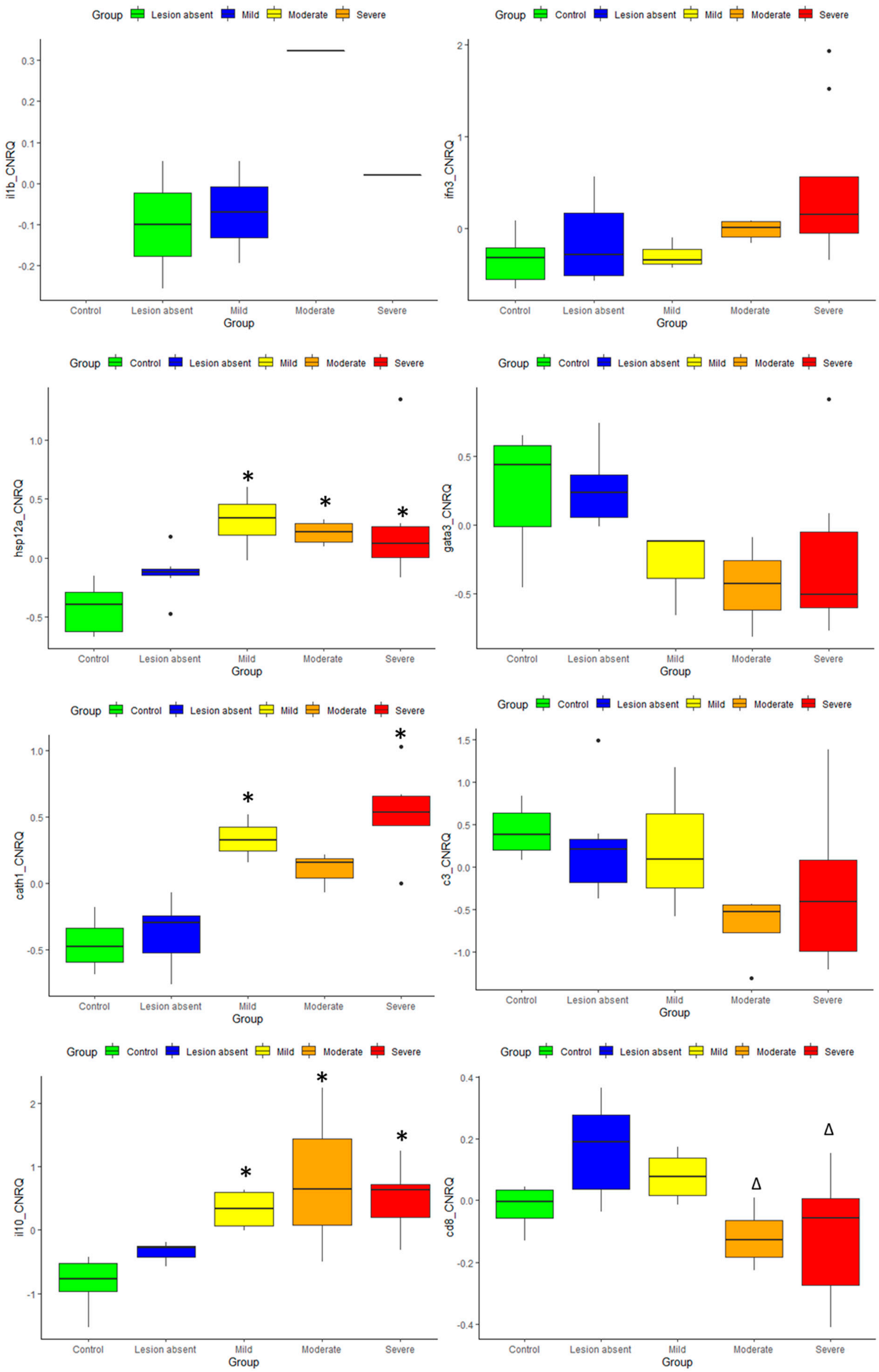
Group Lesion absent Mild Moderate Severe
il1b_CNRQ
0.3
0.2
0.1
0.0
-0.1
-0.2
Control Lesion absent Mild Moderate Severe
Group
Group Control Lesion absent Mild Moderate Severe
ifn3_CNRQ
2
1
0
Control Lesion absent Mild Moderate Severe
Group
Group Control Lesion absent Mild Moderate Severe
hsp12a_CNRQ
1.0
0.5
0.0
-0.5
*
*
*
Control Lesion absent Mild Moderate Severe
Group
Group Control Lesion absent Mild Moderate Severe
gata3_CNRQ
0.5
0.0
-0.5
Control Lesion absent Mild Moderate Severe
Group
Group Control Lesion absent Mild Moderate Severe
cath1_CNRQ
1.0
0.5
0.0
-0.5
*
*
Control Lesion absent Mild Moderate Severe
Group
Group Control Lesion absent Mild Moderate Severe
c3_CNRQ
1.5
1.0
0.5
0.0
-0.5
-1.0
Control Lesion absent Mild Moderate Severe
Group
Group Control Lesion absent Mild Moderate Severe
il10_CNRQ
2
1
0
-1
*
*
*
Control Lesion absent Mild Moderate Severe
Group
Group Control Lesion absent Mild Moderate Severe
cd8_CNRQ
0.4
0.2
0.0
-0.2
-0.4
Δ
Δ
Control Lesion absent Mild Moderate Severe
Group

b) Kidney

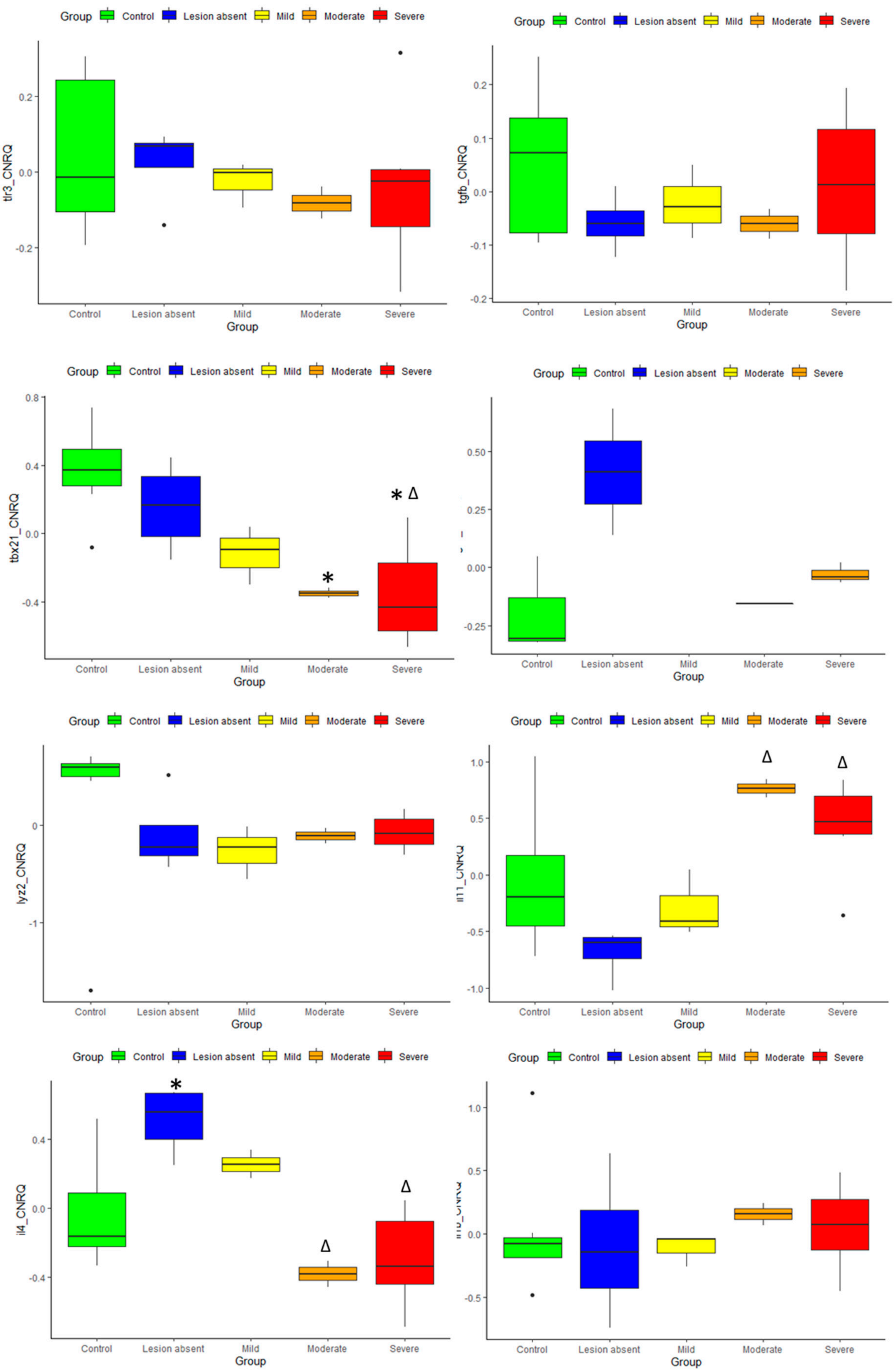
Group Control Lesion absent Mild Moderate Severe
tlr3_CNRQ
tgfb_CNRQ
tbx21_CNRQ
Group Control Lesion absent Moderate Severe
lyz2_CNRQ
il4_CNRQ
Control
Lesion absent
Mild
Moderate
Severe
Group

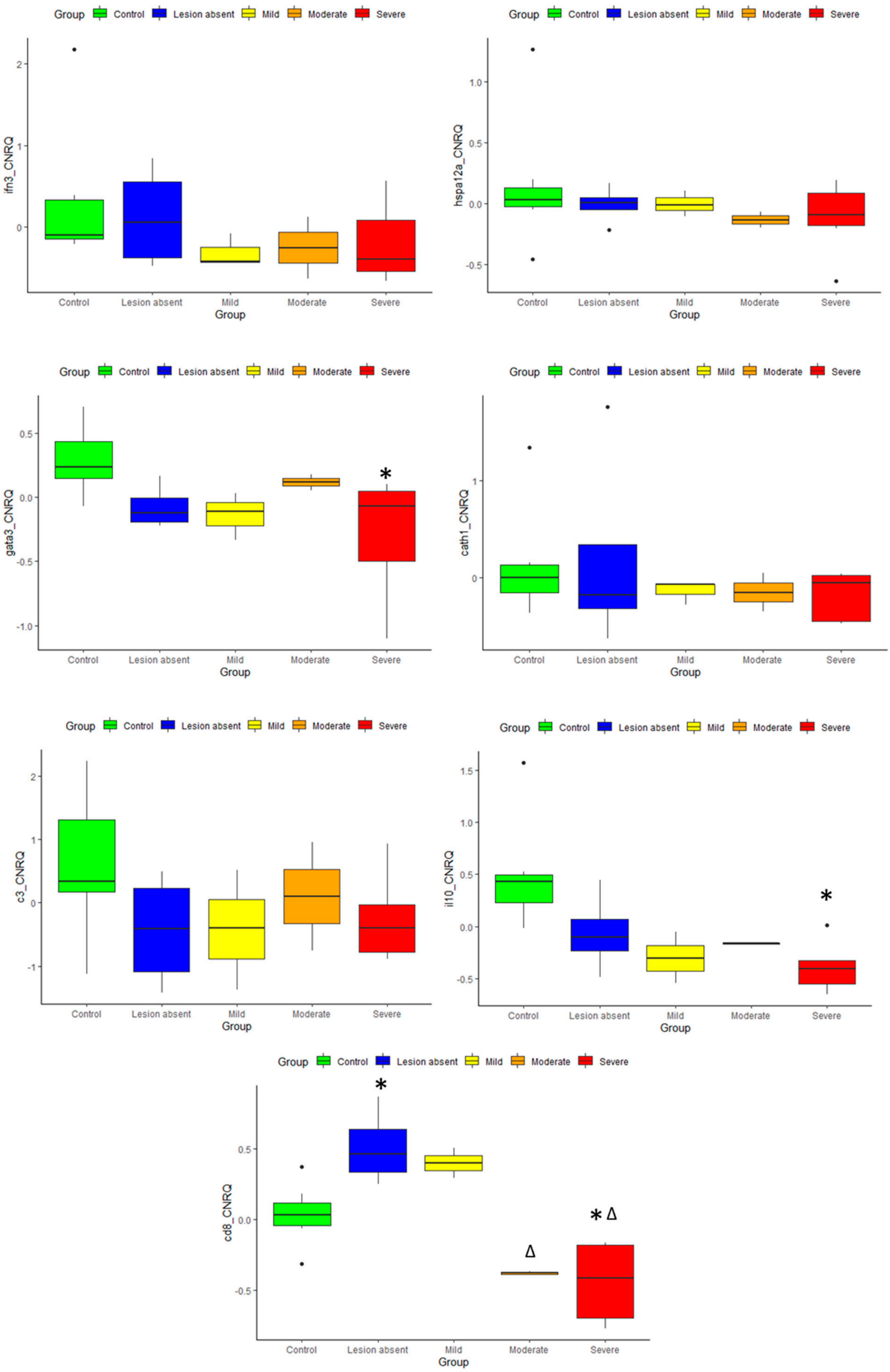
Group Control Lesion absent Mild Moderate Severe
ifn3_CNRQ
2
1
0
Control Lesion absent Mild Moderate Severe
Group
Group Control Lesion absent Mild Moderate Severe
hspa12a_CNRQ
1.0
0.5
0.0
-0.5
Control Lesion absent Mild Moderate Severe
Group
Group Control Lesion absent Mild Moderate Severe
gata3_CNRQ
0.5
0.0
-0.5
-1.0
*
Control Lesion absent Mild Moderate Severe
Group
Group Control Lesion absent Mild Moderate Severe
cath1_CNRQ
1
0
Control Lesion absent Mild Moderate Severe
Group
Group Control Lesion absent Mild Moderate Severe
c3_CNRQ
2
1
0
-1
Control Lesion absent Mild Moderate Severe
Group
Group Control Lesion absent Mild Moderate Severe
il10_CNRQ
1.5
1.0
0.5
0.0
-0.5
*
Control Lesion absent Mild Moderate Severe
Group
Group Control Lesion absent Mild Moderate Severe
cd8_CNRQ
0.5
0.0
-0.5
*
Δ
* Δ
Control Lesion absent Mild Moderate Severe
Group

Figure S1. Relative gene expression of immune related genes in the jaw (a) or the kidney (b) of rainbow trout showing mild (4 fish), moderate (4 fish), or severe (8 fish) clinical signs of cranial maxillary fibrosis syndrome. No-lesion group (7 fish) refers to fish with no apparent lesions sampled in the affected site. The same stock of fish was sampled from a nearby unaffected farm and used as negative controls (7 fish). (*) and (Δ) denotes gene expression significantly different ($p < 0.05$) either from the control group or the no-lesion group respectively. Each bar represents for each fish group the median (cross), upper and lower quartile (box), and upper and lower extreme (line). Single points indicate outliers. CNRQ means calibrated normalized relative quantities in the gene expression, equivalent to the fold change method ($2^{-\Delta\Delta Ct}$). Analysed genes were: *Viperin* (*vig1*), *toll-like receptor 3* (*tlr3*), *transforming growth factor beta* (*tgfb*), *T-bet* (*tbx21*), *immunoglobulin M heavy chain* (*IgM*), *lysozyme II (lyz2)*, *interleukin 11* (*il11*), *interleukin 4/13a* (*il4*), *interleukin 1b* (*il1b*), *type I interferon* 3 (*ifn3*), *heat shock* 70 *kDa protein 12A-like* (*hspa12a*), *GATA-binding protein 3* (*gata3*), *cathelicidin 1* (*cath1*), comp*lement c3* (*c3*), *interleukin 10* (*il10*) and *cluster of differentiation 8* (*cd8a*).

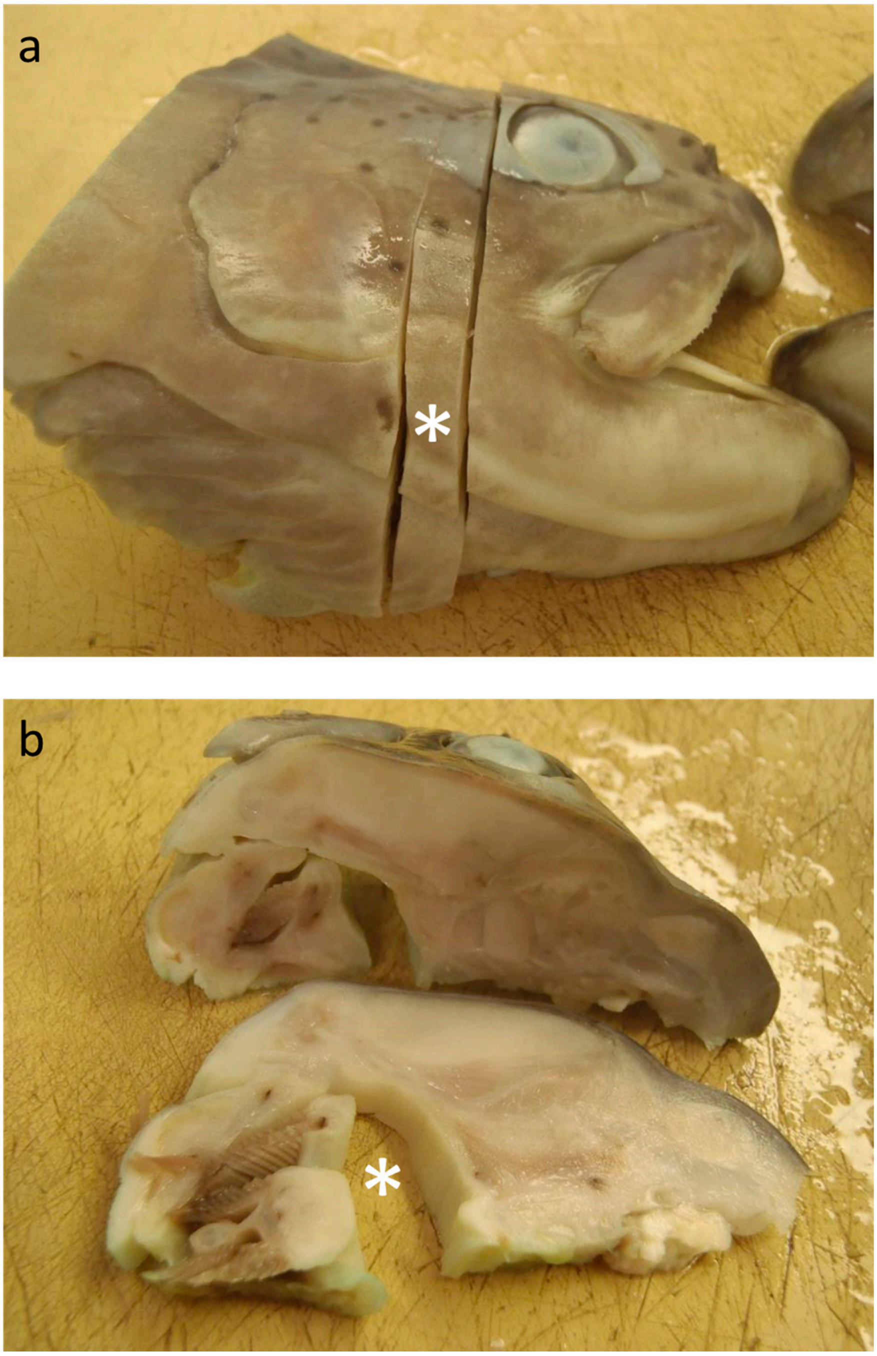

Figure S2. (a, b) Ethanol fixed rainbow trout head showing the location of jaw sections (asterisks) excised for the histopathology and gene expression analysis.

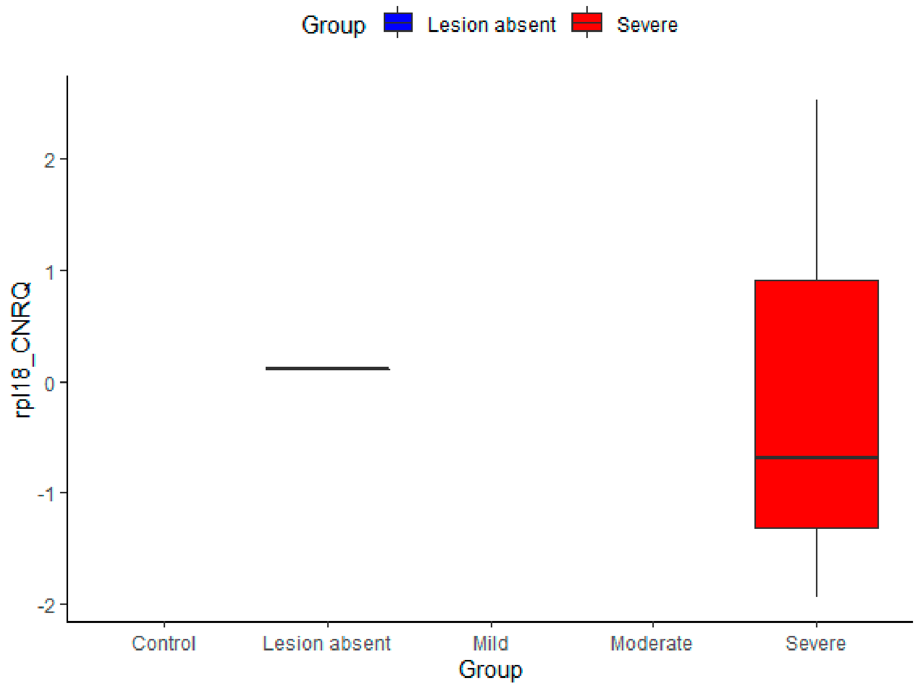

Figure S3. Relative gene expression of *Tetracapsuloides bryosalmonae 60S ribosomal protein l18* (*rpl18*) in the kidney of rainbow trout showing mild (4 fish), moderate (4 fish), or severe (8 fish) clinical signs of cranial maxillary fibrosis syndrome. No-lesion group (7 fish) refers to fish with no apparent lesions sampled in the affected site. A control group (7 fish) was sampled from a nearby unaffected farm. CNRQ means calibrated normalized relative quantities in the gene expression, equivalent to the fold change method ($2^{-\Delta\Delta Ct}$).
